# Ki67 increase after core needle biopsy associated with worse disease outcome in HER2-negative breast cancer patients

**DOI:** 10.1038/s41598-022-25206-1

**Published:** 2023-02-13

**Authors:** Yiwei Tong, Jiangfeng Dai, Jiahui Huang, Xiaochun Fei, Kunwei Shen, Qingmeng Liu, Xiaosong Chen

**Affiliations:** 1 Department of General Surgery, Comprehensive Breast Health Center, Ruijin Hospital, Shanghai Jiao Tong University School of Medicine, 197 Ruijin Er Road, Shanghai, 200025 China; 2 Department of Oncological Surgery, Shaoxing Second Hospital, Shaoxing, Zhejiang China; 3 Department of Pathology, Ruijin Hospital, Shanghai Jiao Tong University School of Medicine, Shanghai, China; 4 Department of Pathology, Shaoxing Second Hospital, Shaoxing, Zhejiang China

**Keywords:** Breast cancer, Prognostic markers

## Abstract

Ki67 would change after core needle biopsy (CNB) in invasive breast cancer. However, whether Ki67 alteration (ΔKi67) influences disease outcomes remains unclear. Here we aim to evaluate the prognostic value of ΔKi67. Patients with paired CNB and open excision biopsy (OEB) samples between January 2009 and June 2016 were retrospectively analyzed. ΔKi67 was calculated as the absolute difference between Ki67 level in CNB and OEB samples, and the median value of 5% was adopted to category patients into high- and low ΔKi67 groups. Disease-free survival (DFS) and overall survival (OS) were compared between different ΔKi67 groups. Overall, 2173 invasive breast cancer patients were included. Median Ki67 was higher in OEB than CNB samples: 25.00% versus 20.00% (*P* < 0.001). Axillary nodal status, STI, histological grading, and molecular subtype were independently associated with ΔKi67 (*P* < 0.05). In the whole population, patients with low ΔKi67 showed superior 5-year DFS (89.6% vs 87.0%, *P* = 0.026), but similar OS (95.8% vs 94.3%, *P* = 0.118) compared to those with high ΔKi67. HER2 status at surgery was the only significant factor interacting with ΔKi67 on both DFS (*P* = 0.026) and OS (*P* = 0.007). For patients with HER2-negative disease, high ΔKi67 was associated with worse 5-year DFS (87.2% vs 91.2%, *P* = 0.004) as well as impaired 5-year OS (93.9% vs 96.8%, *P* = 0.010). ΔKi67 had no significant impact on survival of HER2-positive patients. Ki67 increase after CNB was significantly associated with worse disease outcomes in HER2-negative, but not in HER2-positive patients, which warrants further study.

## Introduction

Core needle biopsy (CNB) is an important tool to ensure a diagnosis of breast cancer and assess biomarkers including estrogen receptor (ER), progesterone receptor (PR), proliferation markers such as Ki67, and human epidermal growth factor receptor 2 (HER2) status before the initiation of breast cancer treatment^1^. Previous studies have shown a high accordance between CNB and open excision biopsy (OEB) in evaluating receptor status^2–6^. However, inevitable discordance can be caused by tumor heterogeneity, CNB quality or quantity, and sample fixation, especially concerning Ki67 expression and subsequent molecular subtype classification^2,3,5,7^.

Intriguingly, several studies found that Ki67 expression level was relatively higher in the OEB specimen compared with paired CNB^3,8–10^. The reasons might be an increasing cancer proliferation of biopsy-driven wound healing stimulation and tumor heterogeneity^8^. Our previous study involving 276 patients revealed several impact factors associated with Ki67 difference between OEB and CNB, including surgery time interval (STI) and molecular subtype^4^.

Ki67 level was an independent predictor for disease-free survival (DFS) and overall survival (OS) in both adjuvant and neoadjuvant settings for early breast cancer patients. Alterations in proliferation index are probably surrogate for changes in tumor growth rate, and might enable the prediction of survival outcomes^11^. A recent meta-analysis found that Ki67 difference after neoadjuvant chemotherapy in breast cancer patients was associated with worse DFS and OS^11^, while there was limited data about the prognostic value of Ki67 difference between OEB and CNB.

Based on above issues, we carried out this study to analyze the accuracy of CNB in determining biomarker status, define factors influencing Ki67 alteration after CNB, and evaluate the potential prognostic value of Ki67 alteration between OEB and CNB in early breast cancer patients.

## Methods

### Patient population

**Figure 1:**
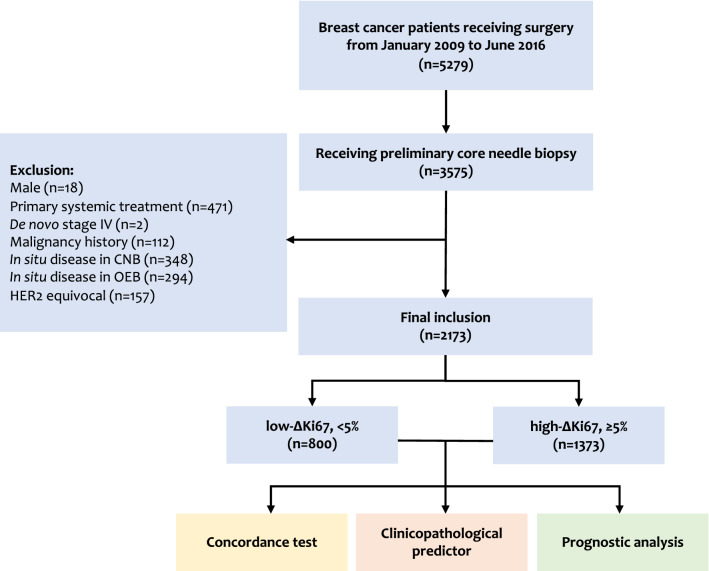
Study flowchart.

Breast cancer patients with paired CNB and OEB samples from January 1st, 2009 to June 30th, 2016 were retrospectively analyzed if they met the following criteria: (1) received both CNB and definitive OEB in Comprehensive Breast Health Center, Ruijin Hospital, Shanghai Jiao Tong University School of Medicine, Shanghai, China or Department of Oncological Surgery, Shaoxing Second Hospital, Shaoxing, Zhejiang, China; (2) female gender; (3) pathological proven invasive carcinoma in both CNB and OEB, with available immunohistochemistry (IHC) and fluorescence in situ hybridization (FISH) results. Patients receiving preoperative therapy, and those with prior or simultaneous malignancies were excluded from the study (Fig. 1). Clinical-pathological characteristics were achieved from Shanghai Jiao Tong University Breast Cancer Database (SJTU-BCDB). This study was approved by the independent Ethical Committees of Ruijin Hospital, Shanghai Jiao Tong University School of Medicine and Ethical Committees of Shaoxing Second Hospital.

### Pathological evaluation and molecular subtypes classification

At least three 14-gauge CNB samples were collected for pathological examination. STI was calculated by the period between the date of OEB and CNB, and categorized into 1–2, 3–4, ≥ 5 days in concordance with our previous work^4^. Tumor pathological evaluation was performed in the Department of Pathology, Ruijin Hospital, Shanghai Jiao Tong University School of Medicine, and Department of Pathology, Shaoxing Second Hospital. CNB samples were collected, and fixed in formalin within 10 min from collection, and the fixation lasted for at least 6 h. OEB specimens were cut at 1 cm intervals, then fixed in formalin within 30 min from collection, and the fixation lasted for at least 24 h to ensure penetration. The volume of formalin was more than 10 times the volume of sample tissue, ensuring that the tumor tissue was completely immersed. CNB and OEB samples were then dehydrated, paraffin-embedded, and sectioned with the same procedures. Paired CNB and OEB were stained using Ventana Autostain System (BenchMark XT, Ventana Medical Systems, Inc., Tucson, AZ). Sections were analyzed and independently reviewed by two of the three experienced pathologists (X. Fei, X. Jin, and Q. Liu). ER or PR positivity were defined as tumors with no less than 1% positive invasive cell nuclear staining. HER2 status was evaluated as described in our previous study according to the ASCO/CAP (American Society of Clinical Oncology/College of American Pathologists) guideline^12,13^. Ki67 level, recorded as the mean percentage of positive invasive tumor cells with any nuclear staining regardless of intensity, was decided by direct counting of 500–2000 tumor cells if homogeneously distributed in tumor sample. In case of heterogeneity, 2000 cells were equally counted in consecutive high-power fields across hot-spot and cold-spot areas for OEB samples, and for CNB samples at least three fields were counted. Ki67 alteration (ΔKi67) was calculated as the absolute difference between Ki67 level in CNB and OEB samples. Hormonal receptor positivity was defined as ER+ or PR+. The cutoff value for Ki67 high expression was 20%, which was described in our previous studies^3,4^. Breast cancer molecular subtype was determined by using the 2013 St. Gallen consensus^14^: Luminal A (Hormonal receptor+/HER2−, Ki67 < 20%), Luminal B-HER2− (ER+/HER2-, Ki67 ≥ 20% or PR < 20%), Luminal B-HER2+ (ER or PR+/HER2+), Triple negative (ER-/PR-/HER2-), and HER2 positive (ER-/PR-/HER2+).

### Adjuvant treatment and follow-up

Adjuvant treatment recommendations were made through multidisciplinary discussion, taking into consideration the combination of CNB and OEB information. Patient follow-up was carried out by specialized breast cancer nurses and assistant in our center. DFS was calculated from the date of surgery to the first proven events including ipsilateral and local/regional recurrence, distant metastasis in any site, second primary tumor, and death of any cause. OS was calculated from the date of surgery till the date of death from any cause. Last follow-up was accomplished by December 2020.

### Statistical analysis

Means, medians, standard deviations, and frequencies were estimated by using standard methods. Wilcoxon signed rank test and Kappa test were applied to compare distribution and concordance rate for pathological factors, respectively, between CNB and OEB. Chi-square test and multivariate logistic regression were adopted to compare the distribution pattern of clinic-pathological factors by ΔKi67. Median ΔKi67 of 5% was adopted to category patients into high- and low ΔKi67 groups. Kaplan–Meier curves were conducted to compare disease outcomes between ΔKi67 groups. Subgroup interaction analysis was conducted by using stratified Mantel–Haenszel test to estimate hazard ratio (HR) with 95% confidence interval (CI). Statistical tests and image construction were accomplished using IBM SPSS version 25 (SPSS, Inc., Chicago, IL) and GraphPad Prism version 8.0 (GraphPad Software, CA, USA). Two-sided *P* values < 0.05 were considered statistically significant.

### Ethics approval and consent to participate

This study was approved by the independent Ethical Committees of Ruijin Hospital, Shanghai Jiao Tong University School of Medicine, and Ethical Committees of Shaoxing Second Hospital. All procedures were in accordance with the ethical standards of the institutional and/or national research committee and with the 1964 Helsinki declaration and its later amendments or comparable ethical standards. Informed consent to participate was obtained from all participants.

## Results

### Baseline characteristics

A total of 2173 patients were included. Baseline patient characteristics were shown in Table 1. The mean age was 56 ± 12.44 (range 23–95) years. Post-menopausal patients accounted for 65.0% of the study population. T_1_ and T_2_ tumors were reported in 53.6% and 43.2% patients, respectively, while 40.7% had positive axillary lymph nodes. The mean STI from CNB to OEB was 5.12 ± 6.30 (range 0–162) days.

**Table 1:** Baseline patient characteristics (*N* = 2173).

Characteristic	*N*	%
**Age, years**	56 ± 12.44 (23–95)	
< 56	1022	47.0
≥ 56	1151	53.0
**Menstrual status**		
Peri/pre-menopause	760	35.0
Post-menopause	1413	65.0
**Breast surgery type**		
Mastectomy	1565	72.0
Lumpectomy	608	28.0
**Clinical tumor size stage**		
T_x_	3	0.1
T_1_	1165	53.6
T_2_	938	43.2
T_3–4_	46	2.1
**Axillary lymph node**		
Negative	1289	59.3
Positive	884	40.7
**Surgery time interval (days)**	5.12 ± 6.30 (0–162)	
1–2	415	19.1
3–4	873	40.2
≥ 5	885	40.7

### Concordance rates of tumor characteristics between CNB and OEB samples

Tumor pathologic characteristics between CNB and OEB samples were presented in Supplementary Table S1. Invasive ductal carcinoma was the most common histologic type, which was found in 90.0% of the CNB samples and 90.5% of the OEB samples. Histologic type was similarly distributed between CNB and OEB (*P* = 0.722), with a concordance rate of 84.33% (κ = 0.573; Table 2). An increased number of grade III tumors was found in OEB samples compared to CNB ones (45.4% vs 34.4%, *P* < 0.001). ER, PR and HER2 status showed good agreement between CNB and OEB, with a concordance rate of 96.23%, 90.98% and 99.13% (κ = 0.906, 0.817 and 0.975; Table 2), respectively. However, median Ki67 level was 25.00% in OEB samples, which was much higher than in CNB samples (20.00%, *P* < 0.001). The proportion of tumors with Ki67 ≥ 20% was 62.6% in OEB samples, which was statistically higher than in CNB ones (51.8%, *P* < 0.001; Supplementary Table S1). The concordance rate for Ki67 was the lowest among common biomarkers (80.53%, κ = 0.607). In addition, the distribution of molecular subtype was also different between CNB and OEB, with more Luminal A tumors by CNB and more Luminal B tumors by OEB (*P* = 0.037). A fair agreement of molecular subtype was found between CNB and OEB in these patients (83.94%, κ = 0.785).

**Table 2:** Concordance between CNB and OEB for pathological factors.

Pathological factors	Concordance rate (%)	Kappa	*P *value
Histologic type	84.33	0.573	< 0.001
Histological grade	95.26	0.741	< 0.001
ER (negative vs positive)	96.23	0.906	< 0.001
PR (negative vs positive)	90.98	0.817	< 0.001
HR (negative vs positive)	96.23	0.905	< 0.001
HER2 (negative vs positive)	99.13	0.975	< 0.001
Ki67 (< 20% vs ≥ 20%)	80.53	0.607	< 0.001
Molecular subtype	83.94	0.785	< 0.001

*CNB* core needle biopsy, *OEB* open excision biopsy, *ER* estrogen receptor, *PR* progesterone receptor, *HR* hormonal receptor, *HER2* human epidermal growth factor receptor-2.

### Impact factors for Ki67 alteration between OEB and CNB

Median Ki67 values at OEB and CNB were 25.00% and 20.00% (*P* < 0.001). The median ΔKi67 of 5% was adopted to classify patients into low- (ΔKi67 < 5%, *N* = 800) and high-ΔKi67 (≥ 5%, *N* = 1373) groups. Supplementary Table S2 showed the univariate analysis results of association between clinic-pathological factors and Ki67 difference. Breast surgery type (*P* = 0.046), clinical tumor size stage (*P* = 0.023), axillary node status (*P* < 0.001), STI (*P* = 0.001), histological grading (*P* < 0.001), ER (*P* = 0.003), PR (*P* < 0.001), HER2 (*P* < 0.001), and molecular subtype (*P* < 0.001) were significantly associated with ΔKi67. Further multivariate analysis demonstrated that axillary node status (*P* = 0.006; Table 3), STI (*P* = 0.001), histological grading (*P* < 0.001), and molecular subtype (*P* < 0.001) were independent impact factors for ΔKi67. Patients with positive lymph nodes (odds ratio [OR] 1.30, 95% CI 1.08–1.58, *P* = 0.006), longer STI (3–4 days vs 1–2 days: OR 1.51, 95% CI 1.18–1.94, *P* = 0.001; ≥ 5 days vs 1–2 days: OR 1.54, 95% CI 1.21–1.97, *P* = 0.001), or higher grade (grade II vs I: OR 2.25, 95% CI 1.58–3.19, *P* < 0.001; grade III vs I: OR 2.64, 95% CI 1.79–3.89, *P* < 0.001) tended to have greater Ki67 alteration after CNB. In addition, compared to patients with other molecular subtypes, those diagnosed with Luminal A tumors at CNB was less likely to experience Ki67 alteration (*P* < 0.001).

**Table 3:** Multivariate analysis of Ki67 alteration and clinic-pathological factors.

Characteristics	OR	95% CI	*P* value
**Breast surgery**			0.510
Mastectomy	1.0		
Lumpectomy	0.93	0.76–1.15	
**Clinical tumor size stage**			0.363
T_1_	1.0		
T_2_	1.15	0.95–1.39	0.146
T_3-4_	1.03	0.55–1.95	0.920
**Node status**			**0.008**
Negative	1.0		
Positive	1.30	1.07–1.57	
**STI, days**			**0.001**
1–2	1.0		
3–4	1.51	1.18–1.94	0.001
≥ 5	1.54	1.21–1.97	0.001
Grade*			**< 0.001**
I	1.0		
II	2.24	1.57–3.18	< 0.001
III	2.61	1.77–3.86	< 0.001
Estrogen receptor*			0.148
Negative	1.0		
Positive	5.38	0.55–52.75	
Progesterone receptor*			0.913
Negative	1.0		
Positive	1.02	0.77–1.33	
HER2*			0.069
Negative	1.0		
Positive	8.70	0.84–89.70	
Molecular subtype*			**0.001**
Luminal A	1.0		
Luminal B-HER2−	1.48	1.17–1.87	0.001
Luminal B-HER2+	2.02	1.42–2.89	< 0.001
HER2 positive	1.60	1.13–2.26	0.010
Triple negative	1.59	1.16–2.18	0.004

Significant values are in bold.

*OR* odds ratio, *CI* confidence interval, *STI* surgery time interval, *HER2* human epidermal growth factor receptor-2.

*Expression status in CNB sample.

### Association of Ki67 alteration with clinical outcomes

**Figure 2:**
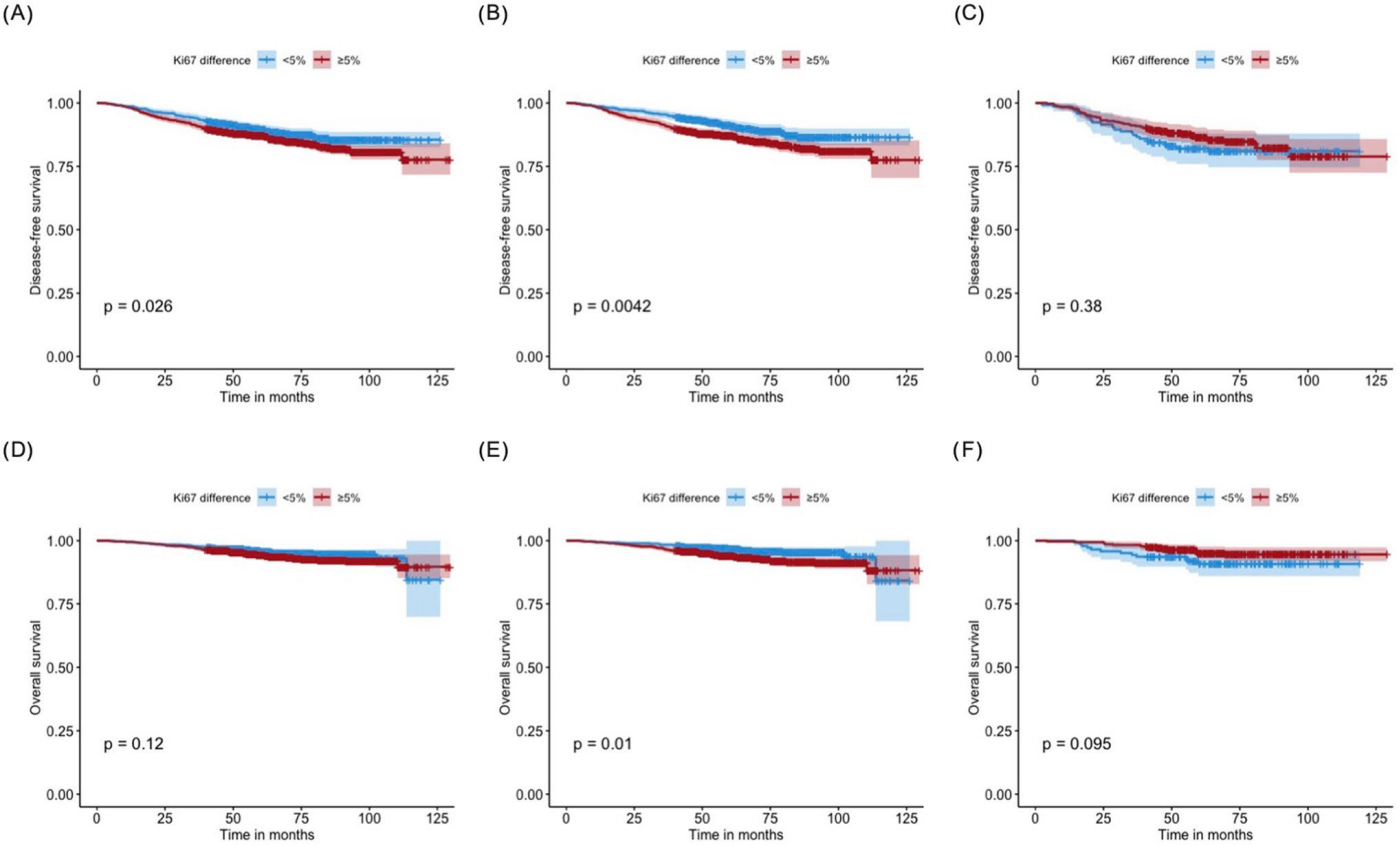
Ki67 difference and disease outcome in breast cancer patients by human epidermal growth factor receptor-2 (HER2) status at surgery. Disease-free survival results for patients with different ΔKi67 (**A**) in the whole population, (**B**) in HER2-negative population, and (**C**) in HER2-positive population. Overall survival results for patients with different ΔKi67 (**D**) in the whole population, (**E**) in HER2-negative population, and (**F**) in HER2-positive population.

At a median follow-up time of 68.5 (12.0–129.7) months, 304 disease-free events were reported, including 36 local recurrences, 83 distant metastases, 22 contralateral breast cancer, 35 s non-breast primary malignancies, 97 breast-specific deaths, and 31 deaths of other causes. Univariate analysis demonstrated that breast surgery type, grade, tumor size stage, lymph node status, ER, PR, and molecular subtype were associated with both DFS and OS (all *P* < 0.05; Supplementary Table S3). In the whole population, patients in the low ΔKi67 group had superior 5-year DFS (89.6% vs 87.0%, *P* = 0.026; Fig. 2A), but similar OS (95.8% vs 94.3%, *P* = 0.118; Fig. 2D) compared to high ΔKi67 group. Regarding adjuvant treatments, adjuvant chemotherapy, radiotherapy, and anti-HER2 target therapies were not associated with disease outcomes, while endocrine therapy was related with DFS (*P* = 0.004) and OS (*P* < 0.001; Supplementary Table S4).

**Figure 3:**
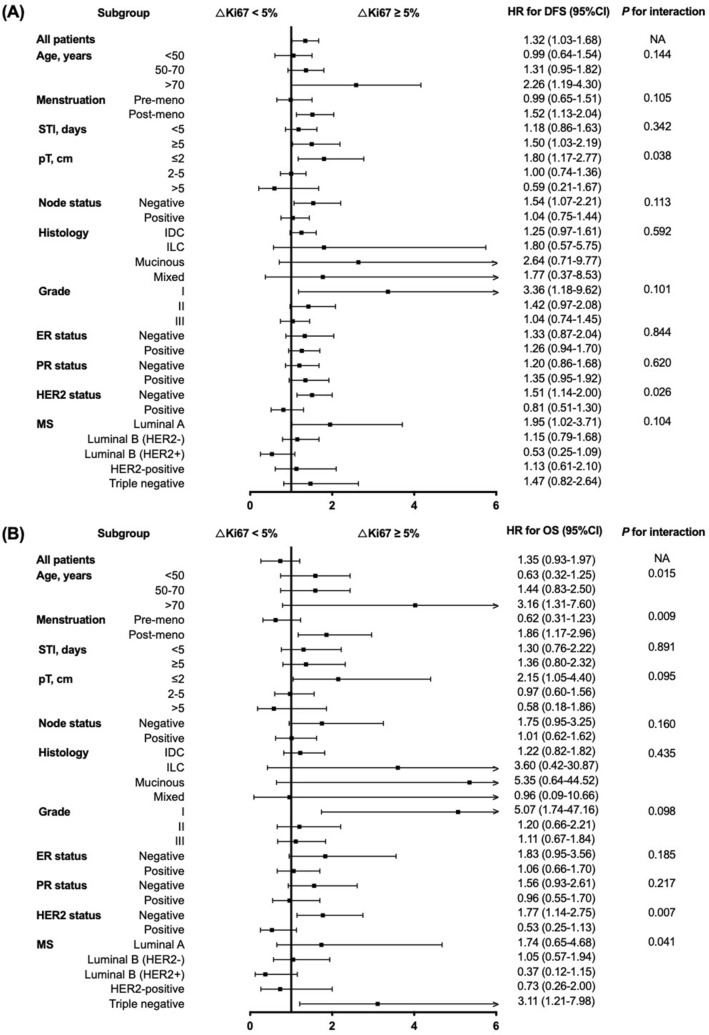
Forest plots and interaction analysis for (**A**) DFS and (**B**) OS in breast cancer patients with different ΔKi67. *DFS* disease-free survival, *OS* overall survival, *HR* hazard ratio, *CI* confidence interval, *NA* not available, *STI* surgery time interval, *IDC* invasive ductal carcinoma, *ILC* invasive lobular carcinoma, *ER* estrogen receptor, *PR* progesterone receptor, *HER2* human epidermal growth factor receptor 2, *MS* molecular subtype.

HER2 status at surgery was the only significant factor identified through subgroup analysis, that interacted with ΔKi67 on both DFS (*P* = 0.026, Fig. 3) and OS (*P* = 0.007). For patients with HER2-negative tumors, high ΔKi67 was associated with worse 5-year DFS (87.2% vs 91.2%, *P* = 0.004; Fig. 2B, Supplementary Table S5) as well as impaired 5-year OS (93.9% vs 96.8%, *P* = 0.010; Fig. 2E, Supplementary Table S5) compared to patients in low ΔKi67 group. However, for patients with HER2-positive disease, 5-year DFS (82.1% vs 86.6%, *P* = 0.379; Fig. 2C, Supplementary Table S6) and 5-year OS (90.8% vs 95.2%, *P* = 0.095; Fig. 2F, Supplementary Table S6) were identical between two groups. In addition, high ΔKi67 after CNB could predict impaired DFS in ER-negative, but not in ER-positive patients. For patients with ER-negative tumors, high ΔKi67 was associated with worse 5-year DFS (82.6% vs 89.2%, *P* = 0.026; Supplementary Fig. S1B) as well as impaired 5-year OS (90.2% vs 96.8%, *P* = 0.009; Supplementary Fig. S1E) compared to patients in low ΔKi67 group.

Furthermore, subgroup analysis revealed that pathological tumor size had a statistically significant interaction with ΔKi67 on DFS (*P* = 0.038; Fig. 3A). In detail, for patients with small tumors, greater ΔKi67 led to significantly impaired DFS (HR 1.80, 95% CI 1.17–2.77), while for patients with tumors more than 5 cm in size, greater ΔKi67 tended to suggest better DFS though not statistically significant (HR 0.59, 95% CI 0.21–1.67). Age (*P* = 0.008; Fig. 3B), menstruation status (*P* = 0.009), and molecular subtype (*P* = 0.041) showed significant interaction with ΔKi67 on OS. For elder, post-menopausal patients, the detrimental effect of greater ΔKi67 was more obvious. When stratified by molecular subtype, greater ΔKi67 was associated with worse DFS in Luminal A (*P* = 0.026; Supplementary Fig. S2A), but not in other molecular subtypes. Meanwhile, greater ΔKi67 showed the most remarkable adverse effect on OS in triple-negative population (HR 3.11, 95% CI 1.21–7.98, *P* = 0.013; Fig. 3, Supplementary Fig. S3E).

## Discussion

In the current study with 2173 invasive breast cancer patients, we demonstrated a high concordance rate for ER, PR, and HER2 status, but a fair agreement for Ki67 and molecular subtype evaluation between CNB and OEB samples. Independent impact factors for Ki67 alteration between OEB and CNB included axillary nodal status, STI, histological grading, and molecular subtype. Moreover, we found that ΔKi67 significantly interacted with HER2 status in prognosis prediction. Greater ΔKi67 was associated with worse disease outcomes in HER2-negative population but not in HER2-positive patients. To our knowledge, this is the largest study to test the concordance rates for receptor factors and Ki67 between CNB and OEB, and to evaluate the prognostic value of Ki67 change after CNB in invasive breast cancer patients with different HER2 status.

CNB, as a minimally invasive method usually obtained by ultrasound or stereotactic guidance, is mandatory to ensure the pathological diagnosis and to evaluate biomarkers before the initiation of any type of treatment for primary breast cancer^1^. Since the 2013 St. Gallen breast cancer consensus, breast cancer has been classified into at least five subtypes: Luminal A, Luminal B-HER2 negative, Luminal B-HER2 positive, triple negative, and HER2 positive based on the IHC results of ER, PR, HER2, and Ki67^14^. The accuracy of CNB in HR evaluation has been reported in several studies, which had a high concordance between CNB and OEB. Our previous meta-analysis including 27 studies demonstrated that a pooled sensitivity of 97.0% and 91.1% for ER and PR testing in CNB^2^. Similarly, Omranipour et al*.* reported that the accuracy of ER and PR testing in CNB was 98% and 93%^15^, which was consistent with You et al.’s study (96.7% for ER and 94.3% for PR testing)^6^. In our study, we included a total of 2173 patients and found the concordance rates for ER, PR, and HER2 status between CNB and OEB were 96.23%, 90.98%, and 99.13% (κ = 0.906, 0.817, and 0.975 respectively), which was similar to previous studies.

Regarding Ki67 testing in CNB, there were only moderate agreement between CNB and OEB in previous reports, which was possibly due to tumor heterogeneity and wound-healing response^3,7,16^. Our previous study showed a concordance rate of 80.4% for Ki67 testing between CNB and OEB, with κ value of 0.600^4^, which was close to the moderate agreement of 80.73% (κ = 0.607) in the current study. A significantly Ki67 value increase was observed in OEB samples compared with CNB. Our previous study enrolling 276 invasive breast cancer patients that STI and molecular subtype were related with Ki67 increase after CNB. After including more patients in the current study, we found that axillary nodal status, STI, histological grading, and molecular subtype were related with Ki67 difference between OEB and CNB. Patients with positive nodes, longer STI, or with higher histological grade, tended to experience Ki67 alteration more often, possibly due to the underestimation caused by minimal sampling at CNB. Tumor size, however, was not independently associated with Ki67 difference between CNB and OEB. In addition, the POETIC trial, including only hormone receptor positive, postmenopausal breast cancer patients, found that Ki67 at baseline, histological grade at baseline, and surgical sample type independently influence Ki67 difference^17^.

Ki67 is a marker for active cell proliferation, which has been proven prognostic in breast cancer. One previous meta-analysis, which included 43 studies, found that Ki67 index was a prognostic factor for both DFS and OS with a large cohort of 15,790 patients^18^. Another meta-analysis including 29 studies also demonstrated that, high Ki67 was associated with impaired DFS and OS in both node-negative and node-positive patients^19^. Ki67 is also predictive for treatment response. Ki67 level decrease during neoadjuvant chemotherapy had been shown to be prognostic for better clinical outcomes^20,21^. A meta-analysis found that an increased Ki67 level after neoadjuvant chemotherapy was associated with worse DFS (HR = 2.13, 95% CI 1.51–3.02)^11^. The IMPACT trial showed that higher Ki67 level after 2 weeks of neoadjuvant endocrine therapy was significantly associated with worse disease outcome (*P* = 0.004), which could better predict survival than Ki67 expression at baseline^22^. In the POETIC trial which enrolled ER positive postmenopausal women^23^, Smith et al*.* showed that Ki67 alteration after two weeks of perioperative endocrine therapy provided additional message for outcome prediction. Those with high Ki67 at baseline, but low Ki67 at 2-week had a significantly reduced risk of recurrence than those who continued to have high Ki67. In the current study, we found that patients with low Ki67 alteration after CNB had a superior 5-year DFS compared to high ΔKi67 population. In spite of the different study designs and enrolled patient characteristics, the above findings suggested that Ki67 alteration could be viewed as a potential marker to predict prognosis as well as treatment response, which should be further validated in clinical studies.

One interesting finding of our study is that the adverse impact of high ΔKi67 after CNB on DFS was only observed in HER2-negative patients but not in HER2-positive ones. Meanwhile, in the POETIC trial, Ki67 after two weeks of perioperative endocrine therapy was remarkably lower in the HER2-negative tumors compared to HER2-positive tumors^23^. These findings suggested that Ki67 alteration might be more susceptible and informative for HER2-negative population. Main possible reason for this lies in that HER2 per se is a more potent biomarker for tumor proliferation. Back in 2003, Tagliabue et al*.* have demonstrated that HER2 overexpression played an essential role in the wound-induced breast cancer proliferation, and the removal of HER2 from the cell membrane led to a prominent decrease of the surgery-induced tumor proliferation^16^. As a result, the effect of Ki67 alteration might be concealed by HER2 overexpression in HER2-positive population. In addition, we also found that high ΔKi67 after CNB could predict impaired DFS in ER-negative, but not in ER-positive patients. Possible explanations may include tumor microenvironment alternation, and immune balance interruption after CNB. Immune cells infiltration, particularly tumor-infiltrating lymphocytes, was associated with disease outcome in breast cancer patients, especially for ER-negative patients, but not for Luminal subtypes^24–27^. Moreover, ER negative tumors had a possible more wound-healing response signature after CNB than ER positive tumors, which may explain the worse prognosis in ER negative patients with high ΔKi67 after CNB. Taken together, we suppose that different cutoffs should be set up for Ki67 alteration in different molecular subtypes, so as to provide more information on prognosis prediction.

There are several limitations in this study. Firstly, given the nature of retrospective single-center design, there might exist selection bias. However, in our clinical practice, nearly all patients with suspicious breast lesion would be recommended to receive CNB before final surgical excision, and both CNB and OEB samples were tested for ER, PR, HER2, and Ki67 biomarkers. Moreover, we included a consecutive cohort of unselected early breast cancer patients with all subtypes of breast cancer in the current study. Thus, we were able to stratify patients into different molecular subtypes, and to conduct further detailed subgroup analysis. Besides, majority of our patients received definitive surgical procedure with a very short interval after CNB, which may make our findings less representative for other centers. Meanwhile, here we applied the median value of Ki67 alteration as cutoff, and different cutoffs in different molecular subtypes should be tested in further studies. Furthermore, it was reported that single-cell RNA-sequencing would provide better profile of intratumoral heterogeneity, which was regrettably not available in our current study. On the other hand, a recent study demonstrated that a combined quantitative measure of biomarkers was a better prognostic factor than categorical combinations as molecular subtypes^28^, which can be validated in further analysis.

## Conclusions

CNB is an important manner to ensure a diagnosis of breast cancer and also evaluate ER, PR, HER2, and Ki67 status before the initiation of breast cancer treatment. Our study, which included a large cohort of 2173 invasive breast cancer patients, has shown a high concordance rate for ER, PR, and HER2 status between CNB and OEB, but a fair agreement for Ki67 and molecular subtype testing. Ki67, as a well-known parameter referring to tumor proliferation, would significantly increase after CNB, which was independently influenced by lymph node status, STI, pathological grading, and molecular subtype. We found that Ki67 increase after CNB was associated with worse DFS and OS only in HER2-negative tumors, but not in HER2-positive tumors, which might be explained by tumor microenvironment alternation, immune balance interruption and wound-healing response signature after CNB.

## Supplementary Information

Supplementary Information.

## Data Availability

The original data including Ki67 data is available upon reasonable request to the corresponding author.

## Abbreviations

CNB: Core needle biopsy
ER: Estrogen receptor
PR: Progesterone receptor
HER2: Human epidermal growth factor receptor 2
OEB: Open excision biopsy
STI: Surgery time interval
DFS: Disease-free survival
OS: Overall survival
IHC: Immunohistochemistry
FISH: Fluorescence in situ hybridization
HR: Hazard ratio
CI: Confidence interval
OR: Odds ratio
