# Supplementary material for: Ki67 increase after core needle biopsy associated with worse disease outcome in HER2-negative breast cancer patients
Source: Sci Rep. 2023 Feb 13;13:2489. doi: 10.1038/s41598-022-25206-1 (PMC9925825; doi:10.1038/s41598-022-25206-1)
Supplement: Supplementary file 1 — Supplementary Information. [file 41598_2022_25206_MOESM1_ESM.docx]

**Supplementary Tables**

**Supplementary Table S1. Tumor characteristics for CNB and OEB results**

| **Characteristics** | **CNB** | **OEB** | ***P*-value** |
| --- | --- | --- | --- |
|  | ***N* (%)** | ***N* (%)** |  |
| **Histologic type** |  |  | 0.722 |
| Invasive ductal carcinoma | 1956 (90.0) | 1966 (90.5) |  |
| Invasive lobular carcinoma | 68 (3.1) | 69 (3.2) |  |
| Mucinous carcinoma | 76 (3.5) | 78 (3.6) |  |
| Mixed carcinoma | 73 (3.4) | 60 (2.8) |  |
| **Histological grade** |  |  | <0.001 |
| I | 168 (7.7) | 120 (5.5) |  |
| II | 1071 (49.3) | 1060 (48.8) |  |
| III | 748 (34.4) | 987 (45.4) |  |
| NA | 186 (8.6) | 6 (0.3) |  |
| **Estrogen Receptor** |  |  | 0.498 |
| Negative | 593 (27.3) | 613 (28.2) |  |
| Positive | 1580 (72.7) | 1560 (71.8) |  |
| **Progesterone Receptor** |  |  | 0.051 |
| Negative | 989 (45.4) | 925 (42.6) |  |
| Positive | 1184 (54.5) | 1248 (57.4) |  |
| **Hormonal receptor** |  |  | 0.587 |
| Negative | 589 (27.1) | 605 (27.8) |  |
| Positive | 1584 (72.9) | 1568 (72.2) |  |
| **HER2** |  |  | 0.488 |
| Negative | 1699 (78.2) | 1680 (77.3) |  |
| Positive | 474 (21.8) | 493 (22.7) |  |
| **Ki67, %** |  |  | <0.001 |
| $<$20 | 1047 (48.2) | 812 (37.4) |  |
| $\geq$20 | 1126 (51.8) | 1361 (62.6) |  |
| **Molecular subtype** |  |  | 0.037 |
| Luminal A | 546 (25.1) | 460 (21.2) |  |
| Luminal B-HER2- | 804 (37.0) | 868 (39.9) |  |
| Luminal B-HER2+ | 234 (10.8) | 240 (11.0) |  |
| HER2-positive | 240 (11.0) | 253 (11.6) |  |
| Triple negative | 349 (16.1) | 352 (16.2) |  |

Abbreviation: CNB, core needle biopsy; OEB, open excision biopsy; NA, not available; HER2, human epidermal growth factor receptor-2.

**Supplementary Table S2. Univariate analysis of Ki67 alteration and clinic-pathological factors**

| **Characteristic** | **Ki67% alteration** | | ***P*-value** |
| --- | --- | --- | --- |
|  | **<5%**  ***N* (%)** | **≥5%**  ***N* (%)** |  |
| **Age, years** |  |  | 0.174 |
| <56 | 361 (35.3) | 661 (64.7) |  |
| ≥56 | 439 (38.1) | 712 (61.9) |  |
| **Menstrual status** |  |  | 0.361 |
| Peri/pre-menopause | 270 (35.5) | 490 (64.5) |  |
| Post-menopause | 530 (37.5) | 883 (62.5) |  |
| **Breast surgery type** |  |  | 0.046 |
| Mastectomy | 556 (35.5) | 1009 (64.5) |  |
| Lumpectomy | 244 (40.1) | 364 (59.9) |  |
| **Clinical tumor stage** |  |  | 0.023 |
| T_x_ | 1 (33.3) | 2 (66.7) |  |
| T_1_ | 462 (39.7) | 703 (60.3) |  |
| T_2_ | 311 (33.2) | 627 (66.8) |  |
| T_3-4_ | 16 (34.8) | 30 (65.2) |  |
| **Axillary lymph node** |  |  | <0.001 |
| Negative | 517 (40.1) | 772 (59.9) |  |
| Positive | 283 (32.0) | 601 (68.0) |  |
| **STI (days)** |  |  | 0.001 |
| 1-2 | 185 (44.6) | 230 (55.4) |  |
| 3-4 | 309 (35.4) | 564 (64.6) |  |
| ≥5 | 306 (34.6) | 579 (65.4) |  |
| **Histologic type*** |  |  | 0.144 |
| IDC | 708 (36.2) | 1248 (63.8) |  |
| ILC | 31 (45.6) | 37 (54.4) |  |
| Mucinous carcinoma | 35 (46.1) | 41 (53.9) |  |
| Mixed carcinoma | 26 (35.6) | 47 (64.4) |  |
| **Histological grade*** |  |  | <0.001 |
| I | 105 (62.5) | 63 (37.5) |  |
| II | 403 (37.6) | 668 (62.4) |  |
| III | 224 (29.9) | 524 (70.1) |  |
| NA | 68 (36.6) | 118 (63.4) |  |
| **Estrogen receptor*** |  |  | 0.003 |
| Negative | 189 (31.9) | 404 (68.1) |  |
| Positive | 611 (38.7) | 969 (61.3) |  |
| **Progesterone receptor*** |  |  | <0.001 |
| Negative | 318 (32.2) | 671 (67.8) |  |
| Positive | 482 (40.7) | 702 (59.3) |  |
| **HER2*** |  |  | <0.001 |
| Negative | 663 (39.0) | 1036 (61.0) |  |
| Positive | 137 (28.9) | 337 (71.1) |  |
| **Molecular subtype*** |  |  | <0.001 |
| Luminal A | 270 (49.5) | 276 (50.5) |  |
| Luminal B-HER2- | 281 (35.0) | 523 (65.0) |  |
| Luminal B-HER2+ | 63 (26.9) | 171 (73.1) |  |
| HER2 positive | 74 (30.8) | 166 (69.2) |  |
| Triple negative | 112 (32.1) | 237 (67.9) |  |

Abbreviation: CNB, core needle biopsy; OEB, open excision biopsy; STI, Surgery time interval; IDC, invasive ductal carcinoma; ILC, invasive lobular carcinoma; NA, not available; HER2, human epidermal growth factor receptor-2.

* Expression status in CNB sample.

**Supplementary Table S3. Univariate analysis of clinic-pathological factors affecting DFS and OS in invasive breast cancer patients**

| **Clinic-pathological factors** | **DFS**  ***P-*value** | **OS**  ***P-*value** |
| --- | --- | --- |
| Age | 0.730 | 0.078 |
| Menopause status | 0.168 | 0.016 |
| Breast surgery type | 0.025 | 0.001 |
| Histologic type* | 0.466 | 0.477 |
| Histological grade* | 0.005 | 0.020 |
| Pathological tumor size | <0.001 | <0.001 |
| Lymph node status | <0.001 | <0.001 |
| Estrogen receptor * | <0.001 | <0.001 |
| Progesterone receptor * | <0.001 | <0.001 |
| HER2 * | 0.250 | 0.980 |
| Molecular subtype * | <0.001 | 0.001 |
| STI | 0.279 | 0.030 |
| Ki67 alteration ** | 0.026 | 0.118 |

Abbreviation: DFS, disease free survival; OS, overall survival; STI, surgery time interval.

* Expression status in radical surgical sample

**Ki67 alteration value = Absolute value of Ki67% in open excision biopsy sample minus Ki67% value in core needle biopsy.

**Supplementary Table S4. Univariate analysis of adjuvant treatments affecting DFS and OS in invasive breast cancer patients**

| **Adjuvant treatments** | **DFS**  ***P-*value** | **OS**  ***P-*value** |
| --- | --- | --- |
| Chemotherapy | 0.268 | 0.902 |
| Radiotherapy | 0.333 | 0.677 |
| Target therapy | 0.913 | 0.064 |
| Endocrine therapy | 0.004 | <0.001 |

Abbreviation: DFS, disease free survival; OS, overall survival.

**Supplementary Table S5. Univariate analysis of clinic-pathological factors affecting DFS and OS in HER2-negative breast cancer patients**

| **Clinic-pathological factors** | **DFS**  ***P-*value** | **OS**  ***P-*value** |
| --- | --- | --- |
| Age | 0.934 | 0.022 |
| Menopause status | 0.283 | 0.004 |
| Breast surgery type | 0.083 | 0.003 |
| Histologic type* | 0.173 | 0.212 |
| Grade* | 0.004 | 0.020 |
| pT | <0.001 | <0.001 |
| Lymph node status | <0.001 | <0.001 |
| Estrogen receptor * | 0.017 | <0.001 |
| Progesterone receptor * | <0.001 | <0.001 |
| Molecular subtype * | <0.001 | <0.001 |
| STI | 0.410 | 0.015 |
| Ki67 difference** | 0.004 | 0.010 |

Abbreviation: DFS, disease free survival; OS, overall survival; BMI, body mass index; STI, surgery time interval.

* Expression status in radical surgical sample

**Ki67 difference value = Absolute value of Ki67% in open excision biopsy sample minus Ki67% value in core needle biopsy.

**Supplementary Table S6. Univariate analysis of clinic-pathological factors affecting DFS and OS in HER2-positive breast cancer patients**

| **Clinic-pathological factors** | **DFS**  ***P-*value** | **OS**  ***P-*value** |
| --- | --- | --- |
| Age | 0.439 | 0.479 |
| Menopause status | 0.361 | 0.711 |
| Breast surgery type | 0.202 | 0.194 |
| Histologic type* | 0.576 | 0.628 |
| Grade* | <0.001 | 0.530 |
| pT | 0.002 | 0.013 |
| Lymph node status | 0.001 | 0.008 |
| Estrogen receptor * | 0.025 | 0.490 |
| Progesterone receptor * | 0.021 | 0.079 |
| Molecular subtype * | 0.017 | 0.443 |
| STI | 0.405 | 0.398 |
| Ki67 difference** | 0.379 | 0.094 |

Abbreviation: DFS, disease free survival; OS, overall survival; BMI, body mass index; STI, surgery time interval.

* Expression status in radical surgical sample

**Ki67 difference value = Absolute value of Ki67% in open excision biopsy sample minus Ki67% value in core needle biopsy.

**Supplementary Figures**


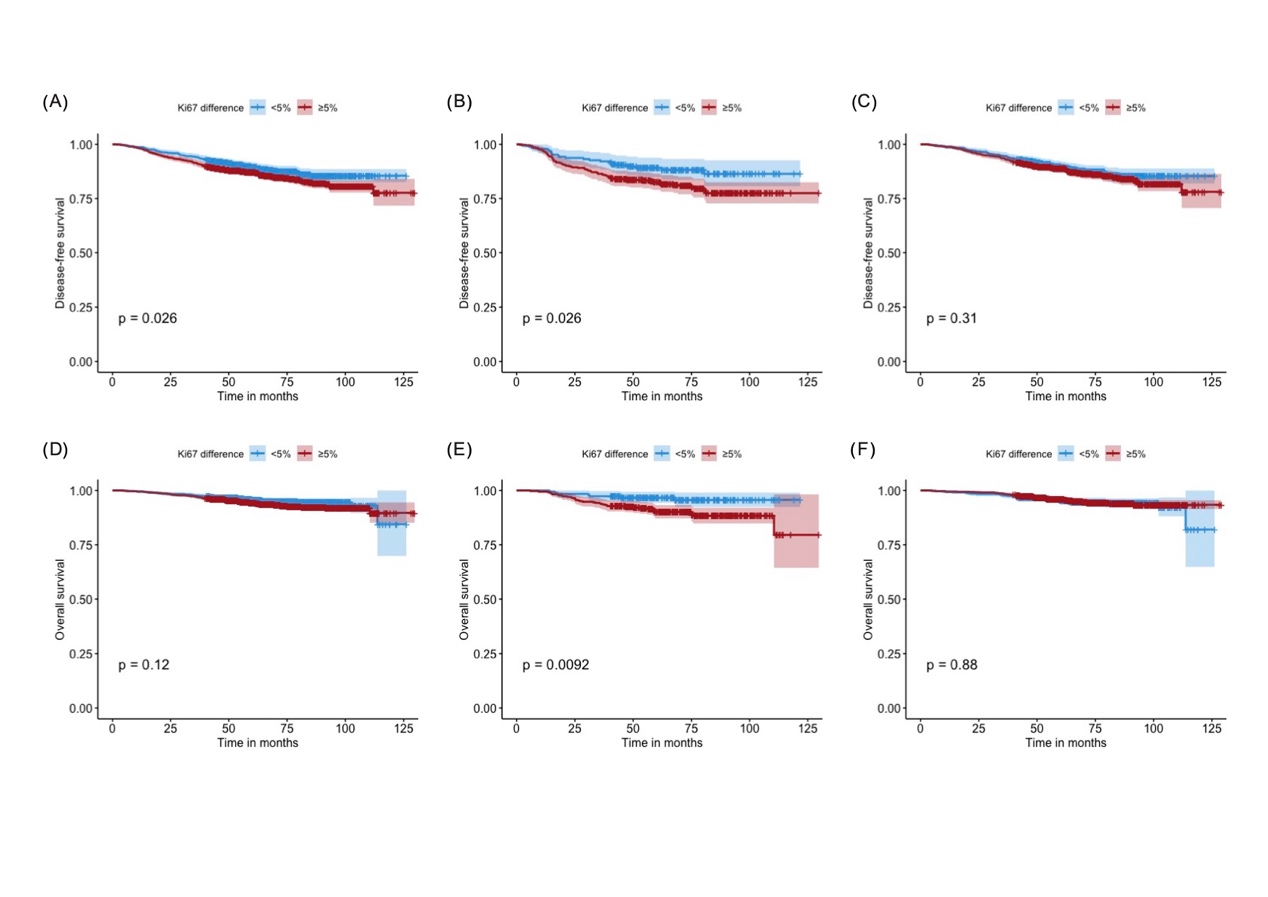


**Supplementary Figure 1. Ki67 difference and disease outcome in breast cancer patients by estrogen receptor (ER) status at core needle biopsy.**

Disease-free survival results for patients with different ΔKi67 (A) in the whole population, (B) in ER-negative population, and (C) in ER-positive population. Overall survival results for patients with different ΔKi67 (D) in the whole population, (E) in ER-negative population, and (F) in ER-positive population.


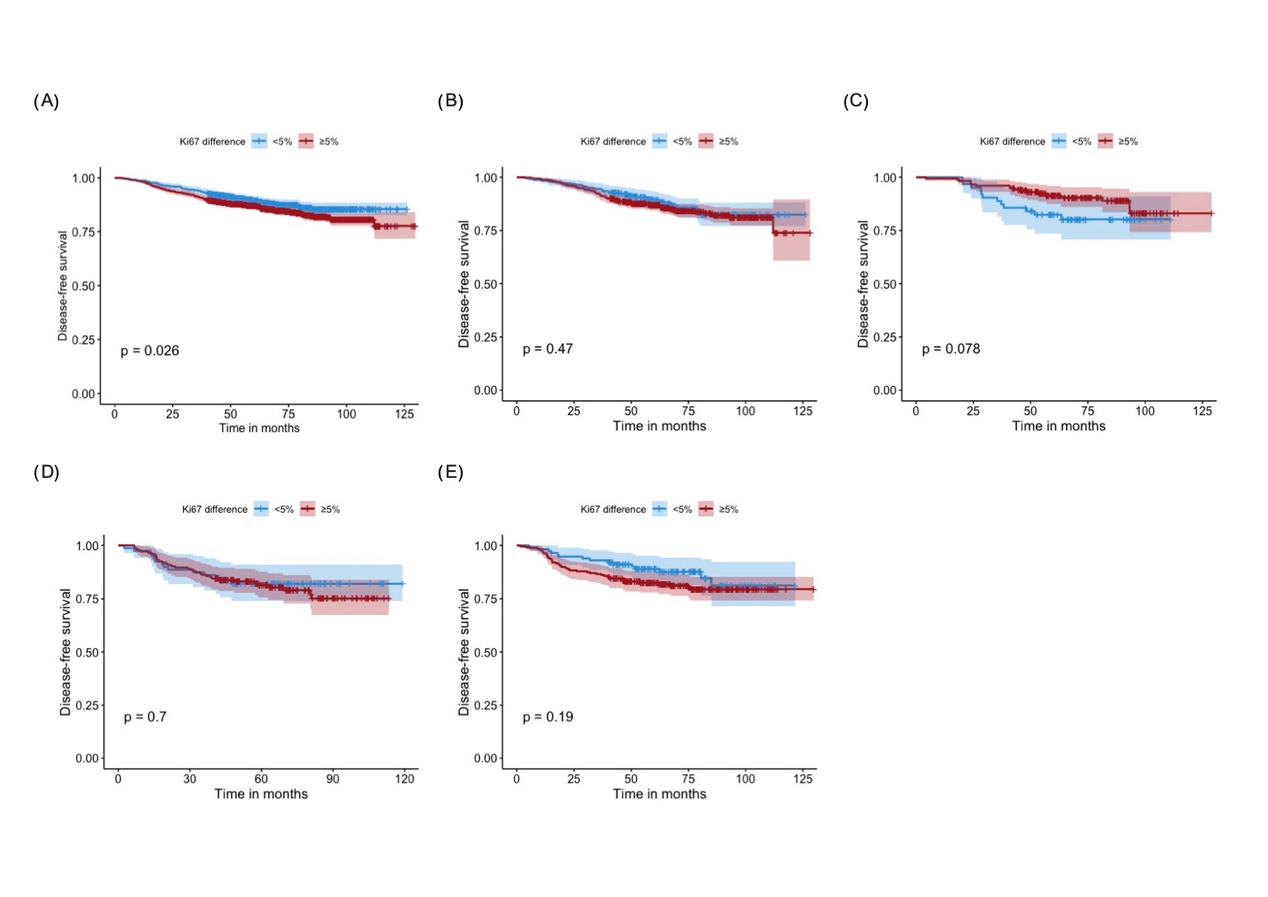


**Supplementary Figure 2. Ki67 difference and DFS in breast cancer patients by molecular subtype.**

DFS for patients with different ΔKi67 in (A) Luminal A, (B) Luminal B HER2-negative, (C) Luminal B HER2-positive, (D) hormone receptor-negative HER2-positive, and (E) triple negative population.

Abbreviations: DFS, disease-free survival; HER2, human epidermal growth factor receptor 2.


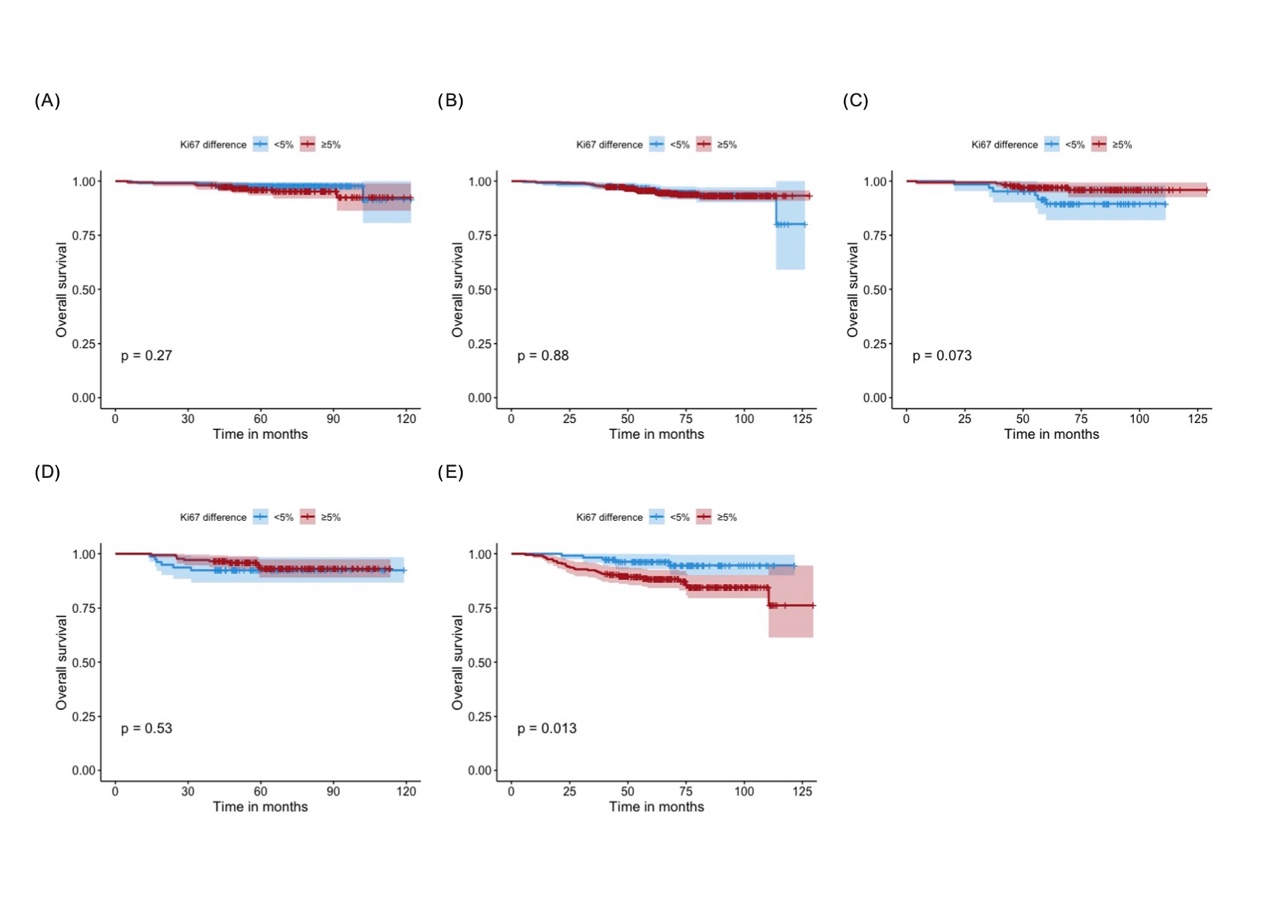


**Supplementary Figure 3. Ki67 difference and OS in breast cancer patients by molecular subtype.**

OS for patients with different ΔKi67 in (A) Luminal A, (B) Luminal B HER2-negative, (C) Luminal B HER2-positive, (D) hormone receptor-negative HER2-positive, and (E) triple negative population.

Abbreviations: OS, overall survival; HER2, human epidermal growth factor receptor 2.
